# Naming diversity in an evolutionary context: Phylogenetic definitions of the *Roucela* clade (Campanulaceae/Campanuloideae) and the cryptic taxa within

**DOI:** 10.1002/ece3.3442

**Published:** 2017-09-20

**Authors:** Andrew A. Crowl, Nico Cellinese

**Affiliations:** ^1^ Florida Museum of Natural History University of Florida Gainesville FL USA; ^2^ Department of Biology Duke University Durham NC USA

**Keywords:** Campanulaceae, clade definition, Mediterranean, *PhyloCode*, phylogenetic nomenclature

## Abstract

In recent times, evolution has become a central tenet of taxonomy, but nomenclature has consistently been decoupled from the tree‐thinking process, often leading to significant issues in reconciling traditional (Linnaean) names with clades in the Tree of Life. Recent evolutionary studies on the *Roucela* clade, a group of endemic plants found in the Mediterranean Basin, motivated the establishment of phylogenetic concepts to formally anchor clade names on the Campanuloideae (Campanulaceae) tree. These concepts facilitate communication of clades that approximate traditionally defined groups, in addition to naming newly discovered cryptic diversity in a phylogenetic framework.

## 1 INTRODUCTION

With the advent of phylogenetic systematics (Hennig, 1950, 1966), tree‐thinking has seized a prominent role in building classifications that more closely reflect the evolutionary history of taxa. However, regardless of how taxa are discovered and diagnosed, biodiversity knowledge is directly or indirectly linked to names. Traditional (Linnaean) names appear to work well for groups that were defined under nonevolutionary frameworks but still approximate clades. However, they may not scale satisfactorily when repurposed for large clades with many nested taxa (e.g., insufficient ranks and/or significant nomenclatural instability due to name changes, among other issues) or to name newly discovered biodiversity below species level (e.g., naming cryptic taxa without destabilizing currently accepted species names). The motivation to develop a phylogenetic system of nomenclature has been well delineated in numerous papers since the early 1990's (de Queiroz & Gauthier, 1990, 1992, 1994), including alternative approaches to naming species (Cellinese, Baum, & Mishler, 2012; Dayrat, Schander, & Angielczyk, 2004).

However, more specifically rationale for generating and making available meaningful phylogenetic definitions (phyloreferences) to (1) reconcile taxonomic names with their concepts; (2) allow data integration on the Tree of Life with confidence; (3) query the Tree of Life without ambiguity and ultimately foster discovery has been covered only in the realm of phyloinformatics (N. Cellinese et al., unpublished).

In this context, we strongly feel that naming biodiversity within a phylogenetic framework is crucial to our goal of communicating taxon concepts. Here, we propose a number of phyloreferences that anchor names to specific parts of the Campanuloideae (Campanulaceae) phylogeny and we follow the general guidelines of the *PhyloCode* (https://www.ohio.edu/phylocode) for the establishment of a clade nomenclature. Importantly, this approach to communicating biodiversity has been increasingly adopted in recent years by many researchers across all different domains of Life (Borchiellini et al., 2004; Cantino et al., 2007; Conrad, Ast, Montanari, & Norell, 2011; Cárdenas, Pérez, & Boury‐Esnault, 2012; Hill et al., 2013; Hundt, Iglésias, Hoey, & Simons, 2014; Joyce, Lyson, & Kirkland, 2016; Li et al., 2016; Mannion, Upchurch, Barnes, & Mateus, 2013; Poe et al., 2017; Rabi, Sukhanov, Egorova, Danilov, & Joyce, 2014; Schoch, 2013; Soltis et al., 2011; Sterli, Pol, & Laurin, 2013; Torres‐Carvajal et al., 2016; Wojciechowski, 2013; Wright, Ausich, Cole, Peter, & Rhenberg, 2017; among others).

Two recent phylogenetic studies have focused on the evolution of the *Roucela* complex (Crowl, Myers, & Cellinese, 2017; Crowl et al., 2015). These groups include small, herbaceous, annual *Campanula* plant species restricted to the Mediterranean Basin and characterized by a dichotomous branching habit and unappendaged calyx lobes (Carlström, 1986; Lammers, 2007). The last available taxonomic revision by Carlström (1986) recognized 12 morphological species, and later Tan and Sorger (1986) added to this complex by describing *C*. *lycica* from Turkey.

This group has historically been very challenging to disentangle morphologically, resulting in its assignment to various ranks, including its own genus distinct from *Campanula* (*Roucela*; Dumortier, 1822), and later demoted to a subgenus of *Campanula* (Damboldt, 1976; Lammers, 2007).

Our recent study used this group as a model for understanding historical drivers of speciation and endemism in the Mediterranean Basin because of its narrow distribution and high level of endemism in this region (Crowl et al., 2015). Most species are narrow endemics to one or few islands primarily in the eastern Mediterranean Basin, with the notable exception of *C. erinus*, which is widespread across the Mediterranean climate zone, from the Arabian Peninsula to Macaronesia, including some coastal regions of North Africa.

In addition to disentangling the complexity of species relationships within this group, we also attempted to understand the processes leading to the peculiar distribution of *C. erinus* compared to its close relatives. Our phylogenetic analyses suggested that the nonmonophyly of *C. erinus* may be due to hybridization and cryptic diversity within this species. In Crowl et al. (2017), we uncover evidence that supports the occurrence of a hybridization event between the tetraploid *C. erinus* and the tetraploid *C. creutzburgii*, leading to an octoploid hybrid taxon that has remained historically hidden within the tetraploid *C. erinus*, mainly because it is morphologically indistinct from its parent. However, not only are these taxa genetically distinct, but they also occupy different geographic ranges. The tetraploid *C. erinus* is exclusive to the western Mediterranean Basin, whereas the octoploid *C. erinus* is found from the Balkans throughout the eastern range, including many islands. The generation of meaningful phylogenetic definitions and assignment of clade nomenclature to these independent entities are important to facilitate communication and query of these taxa and build a more accurate classification.

## 2 THE *ROUCELA* CLADE

**Figure 1:**
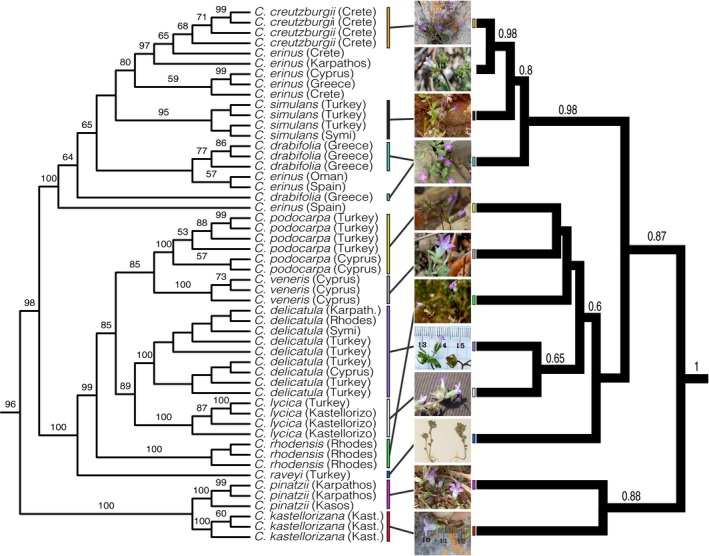
Composition of and phylogenetic relationships within the *Roucela* clade. Results from concatenated RAxML analysis (left) and *BEAST species‐tree analysis (right). Bootstrap support (>50%) and posterior probability values (>0.50) given above branches. Photograph of *Campanula podocarpa* by Charalambos Christodoulou. Remaining photographs by AA Crowl. Redrawn from Crowl et al. (2015)

Several molecular phylogenetic studies (Cellinese et al., 2009; Haberle et al., 2009; Mansion et al., 2012) have consistently recovered a highly supported clade that traditionally included 12 annual *Campanula* species (Campanulaceae) found in the Mediterranean Basin (Carlström, 1986). Traditionally, this group has previously been referred to as the genus *Roucela* (Dumort.) Damboldt, or *Campanula* subg. *Roucela* (Dumort.) Damboldt, or the *Roucela* complex. More recently, Crowl et al. (2015) elucidated the systematics and historical biogeography of this clade (Figure 1), motivating the establishment of a formal phylogenetic definition for this group.

### 2.1 Phylogenetic definition

*ROUCELA* (Dumort.) Damboldt 1976 [A. A. Crowl & N. Cellinese], *nomen cladi conversum*.

#### 2.1.1 Node‐based definition

The least inclusive clade containing *Campanula erinus* L. 1753, *Campanula rhodensis* A. DC. 1830, and *Campanula pinatzii* Greuter & Phitos 1967.

### 2.2 Etymology

The name *Roucela* has previously been used at the rank of genus (*Roucela* Dumort.) and, more recently, subgenus (*Campanula* subg. *Roucela* [Dumort.] Damboldt). Here, we repurpose the name *Roucela* as a clade name to approximate the traditionally defined group named *Campanula* subg. *Roucela* (Dumort.) Damboldt. We prefer to select the name *Roucela* because it is the oldest name by which this group of close relatives has been consistently referred to.

### 2.3 Reference phylogeny

Crowl et al. (2015, figure 2, page 4).

### 2.4 Composition

In the most recent taxonomic revision, Carlström (1986) recognized 12 species in *Campanula* subg. *Roucela* (Dumort.) Damboldt: *C. creutzburgii* Greuter, *C. delicatula* Boiss., *C. drabifolia* Sm., *C. erinus* L., *C. kastellorizana* Carlström, *C. pinatzii* Greuter & Phitos, *C. podocarpa* Boiss., *C. raveyi* Boiss., *C. rhodensis* A. DC., *C. scutellata* Griseb., *C. simulans* Carlström, and *C. veneris* Carlström. More recently, one additional species, *C*. *lycica* was described and added to this group by Tan and Sorger (1986). Although cryptic diversity appears to exist within *Campanula erinus* L., both *C. erinus* lineages were found to fall within this group. According to the phylogenetic analyses of Mansion et al. (2012) and Crowl et al. (2015), *C. scutellata* Griseb. does not appear to belong to this clade.

### 2.5 Diagnostic apomorphies

In addition to molecular synapomorphies, members of the *Roucela* clade are annual *Campanula* species with dichotomous branches and an unappendaged calyx (Carlström, 1986, Lammers, 2007).

### 2.6 Synonyms

None.

### 2.7 General comments on *Roucela*

The group was initially recognized on the basis of morphology: small, dichotomously branched annuals with unappendaged calyx lobes (Carlström, 1986; Lammers, 2007). See Crowl et al. (2015, figure 2) for the primary reference phylogeny. Because *Campanula erinus* L. 1753 (synonym: *Roucela erinus* [L.] Dumort.) was the type species of the previously recognized genus and subgenus, we have included it as an internal specifier in our clade definition.

The distribution of the *Roucela* clade spans the Mediterranean Basin. As traditionally recognized, the most widespread taxon, *C. erinus*, is found from the Azores, southern Europe, northern Africa, and the Arabian Peninsula, an area broadly corresponding to the Mediterranean climate zone but extending as far east as Iran. The remaining species occupy more restricted distributions—many, narrow island endemics in the Aegean Archipelago, western Turkey, and Cyprus.

The phylogenetic analyses of Crowl et al. (2015) included all 13 traditionally recognized species in the *Roucela* complex. Utilizing both plastid and nuclear markers, this study recovered strong support for the monophyly of the group within the broader Campanuloideae clade, with the exclusion of *Campanula scutellata*. Carlström (1986) pointed out the morphological divergence of *C. scutellata* as compared to other species in the group. Phylogenetic analyses have confirmed this observation and suggest this species is more closely related to other annual taxa in the *Megalocalyx* clade (Crowl et al., 2015; Mansion et al., 2012).

Past studies suggest the *Roucela* clade may be closely related to a clade containing Northern African and Western Mediterranean taxa (Cellinese et al., 2009; Haberle et al., 2009), although increased taxon sampling indicates that this clade may also contain Asian campanuloids (Mansion et al., 2012; Crowl et al., 2016).

Within the *Roucela* clade, the geologic history of the eastern Mediterranean appears to have played an important role in the diversification of many species, while the climatic history—specifically, the shift from a subtropical climate—may have adversely affected diversification (see Crowl et al., 2015 for an in‐depth discussion).

## 3 THE *HOLOERINUS* CLADE

**Figure 2:**
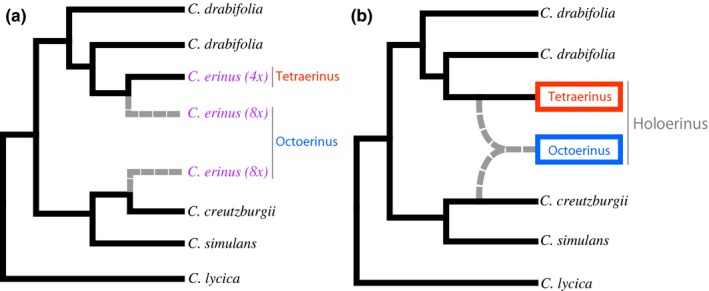
Phylogenetic placement of *Tetraerinus*,* Octoerinus*, and *Holoerinus* within the *Roucela* clade based on results from Crowl et al. (2017). (a) Composition of *Tetraerinus* and *Octoerinus* clades. Due to its hybrid origin, *Octoerinus* is sister to both parental lineages *C. creutzburgii* and *Tetraerinus*. The conflicting placement of this clade is indicated with gray, dashed lines. (b) Composition of the *Holoerinus* clade. The hybrid origin of *Octoerinus* is indicated with gray, dashed lines

A genomic dataset consisting of 130 nuclear loci and near‐complete plastomes across 27 populations of *Campanula erinus* L., spanning its distribution range, provides strong evidence for two lineages within this currently recognized species (Crowl et al., 2017). While a subset of the nuclear genome suggested the octoploid *C. erinus* lineage to be sister to *C. creutzburgii*, phylogenetic analyses of plastomes and other nuclear loci recovered it as sister to the tetraploid *C. erinus* lineage (Figure 2a), rendering the traditionally circumscribed *C. erinus* monophyletic. We, therefore, have chosen to generate a phylogenetic definition for this clade.

Crowl et al. (2017) concluded that the inconsistent placement of the octoploid lineage was the result of a hybridization event in which the tetraploids *C. creutzburgii* and *C. erinus* were the parental lineages. From a phylogenetic perspective, the octoploid lineage is, therefore, sister to both parental lineages (Figure 2a). The phylogenetic definition provided here refers to the octoploid plus tetraploid *C. erinus* clade but does not include *C. creutzburgii* (Figure 2b).

### 3.1 Phylogenetic definition

*HOLOERINUS* A. A. Crowl & N. Cellinese, *nomen cladi novum*.

#### 3.1.1 Branch‐modified node‐based definition

The most inclusive crown clade containing *Campanula erinus* L. 1753 but not *Campanula creutzburgii* Greuter 1967 or *Campanula drabifolia* Sm. 1806.

### 3.2 Etymology

We choose the name *Holoerinus* to indicate the inclusion of both tetraploid and octoploid lineages of *C. erinus*. This name combines the specific epithet of the traditionally recognized species, *Campanula erinus*, with the Greek prefix, *holo*‐ which means “whole.”

### 3.3 Reference phylogeny

Crowl et al. (2017, figure 3, page 919).

### 3.4 Composition

The *Holoerinus* clade is composed of both tetraploid and octoploid lineages within the traditionally recognized *Campanula erinus*, occurring throughout the Mediterranean Basin.

### 3.5 Diagnostic apomorphies

In addition to molecular apomorphies, members of the *Holoerinus* clade are distinguished from other taxa in the *Roucela* clade on the basis of a reduced corolla length (*ca*. 2–5 mm). Bract morphology differentiates the *Holoerinus* clade (bract tooth *ca*. 1–2.5 mm in length) from the closely related *C. creutzburgii* (bract tooth *ca*. 0–0.5 mm in length).

### 3.6 Synonyms

None, although technically the *Holoerinus* clade approximates to the traditionally established *C. erinus*. However, we choose to ignore ranks (specifically, the rank of species in this case) and apply the name *Holoerinus* to the clade that includes all populations of tetraploid and octoploid *C. erinus*.

### 3.7 General comments on *Holoerinus*

The phylogenetic analyses of Crowl et al. (2015) recovered the taxon *Campanula erinus* L. as nonmonophyletic. Statistical support for this result, however, was insufficient to draw meaningful conclusions. Recent phylogenomic analyses of Crowl et al. (2017), which increased both population and genomic sampling, verified this nonmonophyly only in a subset of the genome, but found *C. erinus* to be monophyletic when considering plastome data and a number of nuclear loci. This study concluded that the observed discordance was the result of hybridization, leading to the formation of two cryptic taxa within the traditionally recognized species. Although seemingly indistinguishable on the basis of morphology, populations belonging to these lineages are recognized on the basis of geography and ploidy: western Mediterranean tetraploids and eastern Mediterranean octoploids. The *Holoerinus* clade, as defined here, includes both of these lineages and approximates in content to the traditionally established *C. erinus* (Figure 2).

## 4 THE *TETRAERINUS* CLADE

Phylogenomic analyses of Crowl et al. (2017) consistently recovered strong support for a clade of tetraploid *Campanula erinus* L. populations within the *Holoerinus* clade. This tetraploid lineage, found throughout the western Mediterranean Basin, occurs from the Azores to the Balkans and includes the type specimen of the traditionally established *C. erinus*. The phylogenetic definition presented here includes an herbarium specimen as an internal specifier.

### 4.1 Phylogenetic definition

*TETRAERINUS* A. A. Crowl & N. Cellinese, *nomen cladi novum*.

#### 4.1.1 Apomorphy‐modified node‐based definition

The most inclusive crown clade exhibiting tetraploidy synapomorphic with Crowl #67 [*Campanula erinus* L.], 2 June 2012, Italy: 3 km west of Baia della Zagare on Strada Provinciale N. 53, dirt road just before entrance to tunnel, FLAS 260389.

### 4.2 Etymology

We have combined the traditional specific epithet of *Campanula erinus* with the Latin prefix, *tetra‐* to reflect the ploidal level of this clade.

### 4.3 Reference phylogeny

Crowl et al. (2017, figure 1, page 914). See also Crowl et al. (2017, figure 2, page 919) for results from species‐tree analyses.

### 4.4 Composition

The *Tetraerinus* clade is composed of tetraploid populations of the traditionally recognized species, *Campanula erinus*, occurring in the western Mediterranean Basin from the Balkans to the Azores.

### 4.5 Diagnostic apomorphies

Tetraploidy (chromosome count of 2*n = *28) in addition to other molecular apomorphies.

### 4.6 Synonyms

None.

### 4.7 Comments

Ploidal estimates of Crowl et al. (2017) suggest a strongly supported clade consisting of tetraploid populations (2*n* = 28) distributed throughout the western Mediterranean Basin. A phylogenetic definition has been created to distinguish this clade from a morphologically similar octoploid lineage within the same traditionally recognized species, *C. erinus* (*Holoerinus* clade).

## 5 THE *OCTOERINUS* CLADE

Phylogenomic analyses of Crowl et al. (2017) inferred a hybridization (allopolyploidy) event leading to the formation of an octoploid lineage within the widespread *Holoerinus* clade. To distinguish the clade containing octoploid populations from the morphologically similar tetraploid *Tetraerinus* clade, we provide a phylogenetic definition for this cryptic, octoploid taxon. This definition uses an herbarium specimen as an internal specifier.

### 5.1 Phylogenetic definition

*OCTOERINUS* A. A. Crowl & N. Cellinese, *nomen cladi novum*.

#### 5.1.1 Apomorphy‐modified node‐based definition

The most inclusive crown clade exhibiting octoploidy synapomorphic with Crowl #2 [*Campanula erinus* L.], 4 May 2011, Greece: southern Crete, small canyon 1 km south of Matala, FLAS 240140.

### 5.2 Etymology

We have combined the specific epithet of *Campanula erinus* with the Latin prefix *octo‐* to reflect the octoploid level of this clade,

### 5.3 Reference phylogeny

Crowl et al. (2017, figure 1, page 914). See also Crowl et al. (2017, figure 2, page 918) for results from species‐tree analyses.

### 5.4 Composition

The *Octoerinus* clade is composed of octoploid individuals within the traditionally circumscribed species, *Campanula erinus*. This cryptic taxon is found primarily in the eastern Mediterranean Basin.

### 5.5 Diagnostic apomorphies

Octoploidy (chromosome count of 2*n* = 56), in addition to other molecular apomorphies.

### 5.6 Synonyms

None.

### 5.7 General comments on *Octoerinus*

We generated a phylogenetic definition for *Octoerinus* to distinguish cryptic octoploid and tetraploid taxa within the traditionally established species, *Campanula erinus*. This octoploid appears to be the result of an allopolyploid event in which the tetraploid *Tetraerinus* and tetraploid *C. creutzburgii* are the parental lineages. The phylogenetic analyses of Crowl et al. (2017) showed that populations of different ploidal levels form separate clades, consistent with distinct geographic ranges. Because of the hybrid origin of this lineage, it is sister to both *C. creutzburgii* and the *Tetraerinus* (Figure 2). *Octoerinus* can be distinguished from *Tetraerinus* based on ploidal level: octoploid (2*n* = 56) and tetraploid (2*n* = 28), respectively.

## 6 CONCLUSIONS

During our studies on the evolution of Mediterranean *Campanula* species, we encountered a number of well‐supported clades that also included unknown cryptic diversity due to hybridization and allopolyploidy. Here, we have established clade nomenclature for this group in order to anchor names and concepts to specific parts of the Campanuloideae tree. Clade nomenclature is native to tree‐thinking, and therefore, it is useful for communicating taxa that are discovered in an evolutionary framework and allows for unambiguous assignment of names to nodes on the Tree of Life.

## CONFLICT OF INTEREST

None declared.

## AUTHOR CONTRIBUTIONS

AAC and NC designed and carried out this study. The manuscript was written by both authors.
